# Community Pharmacists’ Knowledge, Perceptions and Practices Regarding Antibiotic Use and Antimicrobial Resistance in Lusaka, Zambia

**DOI:** 10.1155/jotm/9766260

**Published:** 2026-10-11

**Authors:** Martin Kampamba, Wayne Chiweza, Christabel Mwila Hikaambo, Boris Mwanza, Patricia Mbambara, Ann Silwimbwa, Divine Mbambala, Janipher zulu, Jimmy Hangoma

**Affiliations:** ^1^ Department of Pharmacy School of Health Sciences University of Zambia Lusaka Zambia unza.zm; ^2^ School of Pharmacy Eden University Lusaka Zambia; ^3^ Pharmacy Section Health Science Department Evelyn Hone College Lusaka Zambia; ^4^ Department of Pharmacy School of Health Sciences Levy Mwanawasa Medical University Lusaka Zambia cchs.ac.zm

**Keywords:** antibiotic resistance, antibiotic use, antimicrobial stewardship, community pharmacists, dispensing practices

## Abstract

**Background:**

The inappropriate use of antibiotics is a major contributor to antibacterial resistance, which poses a serious threat to global health. Community pharmacists (CPs) play an essential role in promoting antimicrobial stewardship and the prudent use of antibiotics.

**Aims:**

This study evaluated CPs’ antibiotic dispensing practices, knowledge and perceptions of antibiotics and antibiotic resistance.

**Methods:**

This cross‐sectional survey was conducted among 254 CPs in Lusaka using an adapted self‐administered questionnaire. Data were analysed using STATA Version 15.1. Bivariate and multivariable logistic regression analyses identified factors associated with good knowledge and practices regarding antibiotics and antibiotic resistance.

**Results:**

Of the 254 CPs, 206 (81.0%) demonstrated adequate knowledge, 217 (85.4%) had positive perceptions, and 155 (61.0%) exhibited good antibiotic dispensing practices. However, most pharmacists, 169/254 (66.6%), reported that they only sometimes or rarely dispensed antibiotics strictly upon presentation of a prescription, while 233 (91.7%) perceived weak regulations and inadequate monitoring as facilitating nonprescription antibiotic dispensing. In the adjusted analysis, male pharmacists had lower odds of adequate knowledge (adjusted odds ratio [AOR] = 0.3, 95% confidence interval [CI]: 0.14–0.64) but higher odds of good dispensing practices (AOR = 2.3, 95% CI: 1.27–4.11) than females. Part‐time pharmacists also had higher odds of adequate knowledge than full‐time pharmacists (AOR = 1.5, 95% CI: 1.06–2.21).

**Conclusions:**

CPs demonstrated high levels of knowledge, positive perceptions and generally good antibiotic dispensing practices. Nevertheless, frequent dispensing without prescriptions remained common, indicating important gaps in adherence to recommended dispensing standards. Strengthening regulatory oversight, routine monitoring and targeted antimicrobial stewardship interventions is necessary to promote consistent and responsible antibiotic dispensing in community pharmacies.

## 1. Introduction

Antibiotics are essential medications in the fight against bacterial infections, and they play a critical role in maintaining public health [1, 2]. The misuse and overuse of antibiotics have led to the emergence of antibiotic resistance, a global health threat [3]. Community pharmacists (CPs) are often the first point of contact for patients seeking antibiotics, and their antibiotic dispensing practices, knowledge and perceptions of antibiotics and antibiotic resistance are crucial in minimising antibiotic resistance [4, 5].

Inappropriate antibiotic dispensing practices, such as dispensing antibiotics without a valid prescription or for nonbacterial infections, contribute to antibiotic overuse and misuse, accelerating antibiotic resistance [6]. Pharmacists’ knowledge of antibiotics, including their appropriate use, indications, dosages and potential side effects, is crucial for ensuring antibiotics are prescribed and dispensed responsibly [7, 8]. Limited knowledge or misconceptions about antibiotics can lead to inappropriate dispensing practices and contribute to antibiotic resistance [9].

CPs’ perceptions of antibiotic resistance can influence their willingness to engage in antimicrobial stewardship practices, such as counselling patients on appropriate antibiotic use and resisting pressure to dispense antibiotics unnecessarily [10]. Understanding their perceptions can help tailor interventions to address knowledge and attitude gaps [11]. Although pharmacies strive to improve community health, they must also generate profits to sustain their operations. Coupled with weak regulation of antibiotic dispensing, antibiotics may be dispensed without valid prescriptions, thereby contributing to inappropriate dispensing practices and antibiotic resistance [12]. In some situations, patients approach pharmacists directly and request antibiotics, claiming to know their illness [13]. In many low‐ and middle‐income countries, patients cannot afford consultation fees, and long waiting times for appointments often lead pharmacists to dispense antibiotics without a prescription to patients seeking antibiotic treatment [14].

Given their accessibility to patients, CPs can play a vital role in reducing antibiotic resistance by advising against antibiotic use for self‐limiting infections and contributing to antimicrobial stewardship efforts [15]. Pharmacists can help raise awareness of antibiotic resistance among other healthcare professionals and the wider community [16]. Pharmacists should play a central role in antimicrobial stewardship, supported by robust regulatory frameworks and enforcement of prescription‐only antibiotic dispensing policies to promote rational antibiotic use and reduce antimicrobial resistance [2].

In Zambia, AMR has become an increasing public health concern, with inappropriate antibiotic use identified as an important contributor to the growing burden of resistant infections. Recent evidence suggests that although CPs generally demonstrate positive attitudes towards antimicrobial stewardship, inappropriate antibiotic dispensing and gaps in stewardship implementation remain important challenges, highlighting the need to strengthen pharmacists’ role in combating AMR [17].

Lusaka Province provides an important setting for evaluating CPs’ practices because it has the largest concentration of registered community pharmacies and serves the country’s most densely populated urban area. Consequently, dispensing practices in Lusaka have important implications for antibiotic use and antimicrobial stewardship nationally. Although previous studies in Zambia have examined CPs’ knowledge and antimicrobial stewardship practices, evidence remains limited on the contextual factors that influence antibiotic dispensing behaviour. A distinctive contribution of the present study is its assessment of CPs’ self‐reported perceptions of regulatory, commercial, patient‐demand, and health‐system factors influencing antibiotic dispensing practices, alongside their knowledge, perceptions, and practices, and the independent factors associated with these outcomes. This broader assessment provides context for understanding why dispensing behaviour may not always align with knowledge and perceptions and may inform targeted antimicrobial stewardship, regulatory and professional interventions.

The aim of this study was to evaluate CPs’ practices, knowledge and perceptions regarding antibiotics and antimicrobial resistance in Lusaka, Zambia. The findings are expected to provide critical insights into current dispensing practices and inform efforts to strengthen pharmacists’ knowledge and perceptions, thereby supporting targeted interventions to promote rational antibiotic use and combat antimicrobial resistance.

## 2. Materials and Methods

### 2.1. Study Design, Site and Population

This study employed a descriptive cross‐sectional design and was conducted in community pharmacies in Lusaka, Zambia (Figure 1), between October 2023 and May 2024. Lusaka, the capital city of Zambia, hosts over 50% of the country’s registered community pharmacies, making it an appropriate setting for assessing community pharmacy practices [19]. All registered CPs practising in licenced pharmacies and having at least 1 year of professional experience in a community pharmacy setting were included in the study.

**FIGURE 1:**
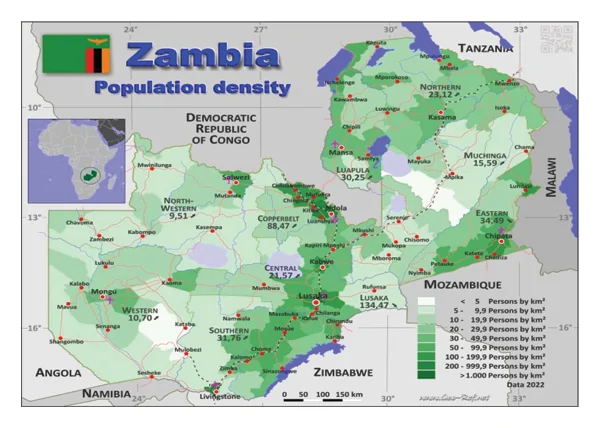
Zambia, a landlocked country in Southern Africa, has an estimated population of approximately 18.4 million, with about 3.61 million residents in Lusaka as of 2022 [18].

### 2.2. Sample Size and Sampling Procedure

A convenience sampling approach was used to recruit registered CPs practising in Lusaka Province from a total population of 690 pharmacists [20, 21]. A target sample size was estimated using Yamane’s formula at a 95% confidence level and a 5% margin of error: where *n* is the sample size, N is the population size (690) and *e* is the margin of error (0.05). The estimated minimum sample size was 254 pharmacists. To account for a potential 10% nonresponse rate, the target sample size was increased to 283 pharmacists. Although convenience sampling was employed because of feasibility, the estimated sample size provided a practical recruitment target and was consistent with sample sizes used in similar studies among CPs.

Eligible pharmacists were identified through visits to community pharmacies across Lusaka Province. Of the 283 pharmacists approached, 265 met the inclusion criteria and were invited to participate using either a self‐administered paper‐based questionnaire or an online questionnaire (Google Forms), according to participant preference. Before participation, the study objectives were explained, and written informed consent was obtained. A total of 254 pharmacists completed the questionnaire, corresponding to a response rate of 95.8% among eligible participants. Completion of the questionnaire required approximately 15–20 min. To prevent duplicate responses, uniquely coded questionnaires were used, and only one response per participant was permitted (Figure 2).

**FIGURE 2:**
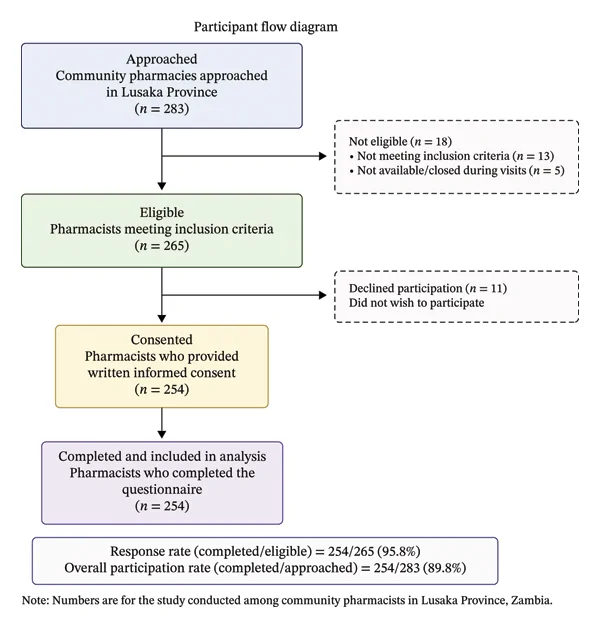
Flow diagram illustrating the recruitment and inclusion of community pharmacists in the study.

### 2.3. Data Collection Instrument

A structured questionnaire was adapted with permission from a previously published study among CPs [22]. The questionnaire comprised five sections. Section 1 collected participants’ sociodemographic characteristics. The instrument was pretested among CPs from 20 community pharmacies, who were excluded from the final study, to assess clarity, relevance, comprehensibility and completeness. Feedback informed minor revisions, and the questionnaire was reviewed by an antimicrobial stewardship expert and two CPs to establish content validity. Section 2 assessed knowledge of antibiotics and antimicrobial resistance using eight items with response options of ‘Yes’, ‘No’ and ‘Not sure’. Correct responses were scored as one, whereas incorrect and ‘Not sure’ responses were scored as zero. Two negatively worded items were reverse coded. The total knowledge score ranged from 0 to 8 and was converted to percentages. Participants scoring ≥ 80% of the maximum attainable score were classified as having good knowledge, while those scoring < 80% were classified as having poor knowledge [17]. Section 3 assessed perceptions regarding antibiotics and antimicrobial resistance using five items with response options of ‘Yes’, ‘No’, and ‘Not sure’. Responses reflecting a positive perception (‘Yes’) were scored as 1, whereas ‘No’ and ‘Not sure’ responses were scored as 0. No perception items required reverse coding. The total perception score ranged from 0 to 5. Using the modified Bloom’s cutoff, scores ≥ 80% (≥ 4/5) were classified as positive perception, while scores < 80% (< 4/5) were classified as negative perception. Section 4 assessed antibiotic dispensing practices using 16 five‐point Likert‐scale items (*always, often, sometimes, rarely* and *never*). Positively worded items were scored from 4 (*always*) to 0 (*never*), whereas negatively worded items were reverse scored from 0 (*always*) to 4 (*never*), ensuring that higher scores consistently reflected better dispensing practices. The total practice score ranged from 0 to 64 and was converted to percentages. Participants scoring ≥ 80% of the maximum attainable score were classified as having good dispensing practices, while those scoring < 80% were classified as having poor dispensing practices [17]. The questionnaire also included items assessing the classes of antibiotics most commonly dispensed without a prescription, which were analysed descriptively. Section 5 assessed CPs’ self‐reported perceptions of factors influencing antibiotic dispensing practices using 22 dichotomous items. Responses were analysed descriptively and presented as frequencies and percentages, and no composite score or classification was generated.

The internal consistency of the scored domains was assessed using Cronbach’s alpha, which yielded a coefficient of 0.79, indicating acceptable reliability.

### 2.4. Data Analysis

Data were entered into Microsoft Excel and exported to Stata Version 15 (StataCorp LLC, College Station, TX, USA) for analysis. Descriptive statistics were used to summarise the data, with categorical variables presented as frequencies and percentages. Pearson’s chi‐square test was used to assess associations between sociodemographic characteristics and knowledge and practice regarding antibiotics and antibiotic resistance. Variables showing an association in the bivariate analysis were further evaluated using bivariate logistic regression to estimate crude odds ratios (CORs) and their corresponding 95% confidence intervals (CIs). Subsequently, multivariable logistic regression analysis was performed to identify factors independently associated with good knowledge and practices. Adjusted odds ratios (AORs) with 95% confidence intervals were reported. The goodness‐of‐fit of the final multivariable logistic regression models was assessed using the Hosmer–Lemeshow test. All statistical tests were two‐sided, and a *p*‐value of less than 0.05 was considered statistically significant.

### 2.5. Ethical Approval

The study was conducted in 2023 following ethical approval from the Excellence in Research Ethics and Science (ERES) Converge Research Ethics Committee (Reference No. 2023‐Aug‐014). Written informed consent was obtained from all participants prior to enrolment. Participants were informed that their participation was voluntary and that they were free to decline participation or withdraw from the study at any time without providing a reason and without incurring any penalty or loss of benefits. Confidentiality and anonymity were maintained throughout the study.

## 3. Results

### 3.1. Sociodemographic Information of CPs According to Antibiotic Dispensing Practices

The study assessed various demographic characteristics of 254 CPs in Lusaka and their antibiotic dispensing practices. Results indicated a significant gender difference, with male pharmacists demonstrating better practices than females [97/138 (70.3%) vs. 58/116 (50.0%), *p* = 0.001]. Age was not significantly associated with dispensing practices; good practices were reported among 75/116 (64.7%) pharmacists aged 21–30 years and 80/138 (58.0%) aged ≥ 31 years (*p* = 0.277). Educational qualification also showed no significant association with dispensing practices (*p* = 0.987). Marital status was significantly associated with practices, with single pharmacists demonstrating better practices than married pharmacists [119/179 (66.5%) vs. 36/75 (48.0%), *p* = 0.006]. Years in practice showed no significant association, although pharmacists with ≥ 6 years of experience had a higher proportion of good practices than those with 1–5 years [44/66 (66.7%) vs. 111/188 (59.0%), *p* = 0.275]. Status at the pharmacy was significantly associated with dispensing practices; part‐time pharmacists had the highest proportion of good practices [94/140 (67.1%)], followed by full‐time pharmacists [56/95 (58.9%)] and store owners [5/19 (26.3%); *p* = 0.002] (Table 1).

**TABLE 1:** Sociodemographic characteristics of community pharmacists according to antibiotic practices (*n* = 254).

Demographic	Variable	Total population *N* = 254	%	Poor practices *N* = 99 (39.0%)	Good practices *N* = 155 (61.0%)	*p*‐value
Gender	Male	138	54.3	41 (29.7)	97 (70.3)	0.001
	Female	116	45.7	58 (50.0)	58 (50.0)	

Age group (years)	21–30	116	45.7	41 (35.3)	75 (64.7)	0.277
	≥ 31	138	54.3	58 (42.0)	80 (58.0)	

Latest educational qualification	BPharm	231	90.9	90 (39.0)	141 (61.0)	0.987
	Higher qualification	23	9.1	9 (39.1)	14 (60.9)	

Marital status	Married	75	29.5	39 (52.0)	36 (48.0)	0.006
	Single	179	70.5	60 (33.5)	119 (66.5)	

Years in practice	1–5	188	74.0	77 (41.0)	111 (59.0)	0.275
	≥ 6 years	66	26.0	22 (33.3)	44 (66.7)	

Status at the pharmacy	Part‐time	140	55.1	46 (32.9)	94 (67.1)	0.002
	Full‐time	95	37.4	39 (41.1)	56 (58.9)	
	Store owner	19	7.5	14 (73.7)	5 (26.3)	

### 3.2. Knowledge of CPs on Antibiotics and Antibiotic Resistance

All 254 participants had heard about antibiotic resistance and agreed that antibiotic resistance can facilitate the spread of infections because of the reduced effectiveness of standard treatment. Overall, 206/254 (81.0%) participants demonstrated adequate knowledge of antibiotics and antibiotic resistance (Figure 3). Only 12/254 (4.7%) incorrectly agreed that antibiotics can treat all infections, including viral and fungal infections, while 34/254 (13.4%) incorrectly agreed that antibiotics can be used as first‐line treatment for colds, coughs, sore throats and uncomplicated malaria. Most participants, 239/254 (94.1%), correctly reported that antibiotics are prescription‐only medicines, while 240/254 (94.5%) recognised that irrational antibiotic use increases the occurrence of antibiotic resistance. Furthermore, 248/254 (97.6%) correctly defined antibiotic resistance as the loss of bacterial sensitivity to an antibiotic, and all participants correctly identified an antibiotic as a drug that prevents or kills bacterial growth (Table 2).

**FIGURE 3:**
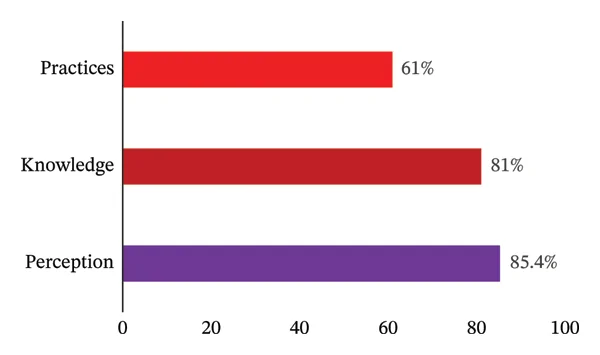
Overall knowledge, perceptions and antibiotic dispensing practices among community pharmacists in Lusaka, Zambia (*n* = 254).

**TABLE 2:** Knowledge of community pharmacists on antibiotics (*n* = 254).

S/N	Questions	Yes (%)	NO (%)	Unsure (%)
1	An antibiotic is a drug that prevents or kills the growth of bacteria	254 (100.0)	0 (0)	0 (0)
2	Antibiotics can be used to treat all infections, including viral and fungal infections.	12 (4.7)	238 (93.7)	4 (1.6)
3	Antibiotics can be used as a first‐line drug in the treatment of cold, cough, sore throat and uncomplicated malaria.	34 (13.4)	220 (86.6)	0 (0)
4	In Zambia, antibiotics are prescription‐only medicines (POM), and there are rules and regulations guiding their sale and dispensing	239 (94.1)	15 (5.9)	0 (0)
5	Have you heard about antibiotic resistance?	254 (100.0)	0 (0)	0 (0)
6	Irrational use of antibiotics increases the occurrence of antibiotic resistance	240 (94.5)	14 (5.5)	0 (0)
7	Antibiotic resistance is a phenomenon where a bacterium loses its sensitivity to an antibiotic.	248 (97.6)	0 (0)	6 (2.4)
8	Antibiotic resistance can lead to the spread of infections due to the reduced effectiveness of standard treatment	254 (100)	0 (0)	0 (0)

*Note:* correct answer = 1; incorrect or ‘Not sure’ = 0. Items 2 and 3 were reverse coded before computation of the total knowledge score.

### 3.3. Perceptions of CPs on Antibiotics and Antibiotic Resistance

As shown in Figure 3, the majority of CPs, 217/254 (85.4%), demonstrated a positive overall perception regarding antibiotic use and antimicrobial resistance (Figure 3). All respondents, 254/254 (100.0%), believed that inadequacies in healthcare facilities in Zambia could influence patients to self‐medicate with antibiotics. Furthermore, 248/254 (97.6%) agreed that antibiotic resistance could jeopardise advancements in modern healthcare if adequate measures were not implemented, while 245/254 (96.5%) believed that inappropriate dispensing of antibiotics could facilitate antibiotic resistance. Most respondents, 240/254 (94.5%), acknowledged that antibiotics can cause side effects or allergic reactions, and 217/254 (85.4%) agreed that culture and sensitivity testing is necessary before recommending antibiotics to patients (Table 3).

**TABLE 3:** Perceptions of community pharmacists on antibiotics and antibiotic resistance.

S/N	Questions	Yes (%)	No (%)	Unsure (%)
1	Do you think inappropriate dispensing of antibiotics to patients can facilitate antibiotic resistance?	245 (96.5)	9 (3.5)	0 (0)
2	Do you think the inadequacies in healthcare facilities in Zambia can influence customers’ self‐medication?	254 (100)	0 (0)	0 (0)
3	Do you think it is necessary to conduct culture and sensitivity tests before recommending antibiotics to patients?	217 (85.4)	37 (14.6)	0 (0)
4	Do you think antibiotic resistance can jeopardise advancements in modern healthcare if adequate measures are not put in place?	248 (97.6)	6 (2.4)	0 (0)
5	Do you think antibiotics have side effects or can cause allergic reactions in patients	240 (94.5)	14 (5.5)	0 (0)

### 3.4. Antibiotics Dispensing Practices by CPs

As shown in Figure 3, the majority of CPs, 155/254 (61.0%), demonstrated good overall practices regarding antibiotic use and antimicrobial resistance. The survey showed that 164/254 (64.6%) pharmacists reported that they sometimes dispensed antibiotics only when accompanied by a prescription, while 61/254 (24.0%) often did so and only 24/254 (9.4%) always followed this practice. Most respondents reported that patients always purchased antibiotics without prescriptions (151/254 (59.4%)). When antibiotics were requested without a prescription, 135/254 (53.1%) pharmacists sometimes asked about their intended use, while 113/254 (44.5%) often did so. Only 47/254 (18.5%) reported that patients always sought their advice before purchasing antibiotics. Regarding appropriate practices, 156/254 (61.4%) pharmacists always updated their knowledge of antibiotics and antibiotic resistance, while 152/254 (59.8%) always counselled patients on the correct dose and duration after dispensing antibiotics (Table 4).

**TABLE 4:** Antibiotic dispensing practices by community pharmacists (*n* = 254).

Code	Practice item	*Always*	*Often*	*Sometimes*	*Rarely*	*Never*
a	A pharmacist must dispense antibiotics only with a prescription	24 (9.4)	61 (24.0)	164 (64.6)	5 (2.0)	0 (0)
b	Patients buy antibiotics without a prescription	151 (59.4)	50 (19.7)	23 (9.1)	10 (3.9)	20 (7.9)
b	Patients buy partial quantities of prescribed antibiotics	33 (13.0)	34 (13.4)	136 (53.5)	27 (10.6)	24 (9.5)
a	Ask why patients request partial quantities	16 (6.3)	74 (29.2)	155 (61.0)	9 (3.5)	0 (0)
a	Counsel on dose and duration after dispensing	152 (59.8)	85 (33.5)	17 (6.7)	0 (0)	0 (0)
a	Ask for intended use when antibiotics are requested without prescription	6 (2.4)	113 (44.5)	135 (53.1)	0 (0)	0 (0)
a	Patients seek advice before purchasing antibiotics	47 (18.5)	99 (39.0)	103 (40.5)	5 (2.0)	0 (0)
b	Suggest antibiotics to unwell patients	26 (10.2)	70 (27.6)	114 (44.9)	39 (15.3)	5 (2.0)
a	Advise patients to complete the antibiotic course	37 (14.5)	68 (26.8)	149 (58.7)	0 (0)	0 (0)
a	Update knowledge on antibiotics and resistance	156 (61.4)	63 (24.8)	35 (13.8)	0 (0)	0 (0)
b	Receive incentives for dispensing particular brands	23 (9.1)	74 (29.1)	36 (14.2)	57 (22.4)	64 (25.2)
a	Ask about medical history before dispensing	18 (7.1)	5 (2.0)	123 (48.4)	108 (42.5)	0 (0)
a	Explain drug interactions before dispensing	68 (26.8)	80 (31.5)	106 (41.7)	0 (0)	0 (0)
b	Dispense more than the recommended quantity	47 (18.5)	36 (14.2)	8 (3.1)	40 (15.8)	123 (48.4)
b	Dispense despite suspected antibiotic misuse	31 (12.2)	22 (8.7)	19 (7.5)	39 (15.3)	143 (56.3)
b	Prescribe and dispense antibiotics after patient complaints	96 (37.8)	44 (17.3)	60 (23.6)	14 (5.5)	40 (15.8)

*Note:* a: *always* = 4, *often* = 3, *sometimes* = 2, *rarely* = 1, *never* = 0 (scoring points). b: always = 0, *often* = 1, sometimes = 2, *rarely* = 3, *never* = 4 (scoring points).

### 3.5. CPs’ Self‐Reported Perceptions of Factors Influencing Antibiotic Dispensing Practices

Most pharmacists, 233/254 (91.7%), agreed that weak regulations and inadequate monitoring contributed significantly to pharmacists’ freedom to dispense antibiotics without prescriptions in Zambia. More than half, 175/254 (68.9%), reported that selling antibiotics generated greater profits. In addition, 184/254 (72.4%) believed that patients’ lack of confidence and trust in physicians was one reason they obtained antibiotics from community pharmacies (Table 5).

**TABLE 5:** Community pharmacists’ self‐reported perceptions of factors influencing antibiotic dispensing practices (*n* = 254).

S/N	Questions	Yes (%)	No (%)	Not sure (%)
1	I dispense antibiotics without prescription so as to meet a given target.	48 (18.9)	206 (81.1)	0 (0)
2	I dispense antibiotics basically to promote some brands.	16 (6.3)	232 (91.3)	6 (2.4)
3	Pressures from patients usually make me dispense antibiotics to them if they do not have prescription.	67 (26.4)	184 (72.4)	3 (1.2)
4	Will turning down customers’ requests for antibiotics have a negative impact on your business?	205 (80.7)	41 (16.1)	8 (3.2)
5	Does the fear of losing your customers make you dispense antibiotics without a prescription	54 (21.3)	200 (78.7)	0 (0)
6	Does the fear of losing your licence refrain you from dispensing antibiotics without prescription?	60 (23.6)	194 (76.4)	0 (0)
7	Are you aware of the potential side effects or allergies customers might be predisposed to when you dispense antibiotics to them without prescription?	221 (87.0)	33 (13)	0 (0)
8	Does the sale of antibiotics boost your daily income at your pharmacy?	25 (9.8)	229 (90.2)	0 (0)
9	Do you readily dispense antibiotics to regular customers without prescription?	33 (13.0)	221 (87.0)	0 (0)
10	Do incentives from medical sales representatives encourage you to sell antibiotics to patients?	70 (27.6)	184 (72.4)	0 (0)
11	Do you dispense antibiotics to children/adults who present any viral infection?	45 (17.7)	209 (82.3)	0 (0)
12	Do you make more profits when you sell antibiotics to patients?	175 (68.9)	69 (27.2)	10 (3.9)
13	Weak regulations and less monitoring play significant roles in pharmacists’ freedom to dispense antibiotics without prescription in Zambia.	233 (91.7)	18 (7.1)	3 (1.2)
14	The sales of antibiotics without prescription at the pharmacy are cost‐effective and time‐saving for the consumers.	80 (31.5)	174 (68.5)	0 (0)
15	Do you still go ahead to dispense antibiotics to patients even if you are not sure if the illness is caused by bacteria or virus?	14 (5.5)	240 (94.5)	0 (0)
16	If a patient’s disease condition is severe, do you dispense antibiotics to them than recommend them to go to the hospital?	25 (9.8)	229 (90.2)	0 (0)
17	Would you prefer to create time to advise patients on antimicrobial resistance than to dispense antibiotics?	249 (98.0)	5 (2.0)	0 (0)
18	Do you request for laboratory reports from the patients before dispensing antibiotics?	196 (77.2)	58 (22.8)	0 (0)
19	Do you readily dispense antibiotics to patients because you are more knowledgeable about drugs?	120 (47.2)	134 (52.8)	0 (0)
20	Patients’ lack of confidence and trust in physicians can be one of the reasons why they come to the pharmacy for their antibiotics.	184 (72.4)	70 (27.6)	0 (0)
21	Do you get pressured by your employer to dispense antibiotics without prescription in order to make more sales?	35 (13.8)	219 (86.2)	0 (0)
22	Do you stylishly force patients to buy antibiotics even if they do not necessarily need them?	0 (0)	243 (95.7)	11 (4.3)

### 3.6. Unadjusted and Adjusted Logistic Regression Analysis of Factors Associated With Knowledge and Practices Regarding Antibiotics and Antibiotic Resistance

In the unadjusted analysis, male pharmacists had lower odds of adequate knowledge (COR = 0.2, 95% CI: 0.12–0.52) but higher odds of good practices (COR = 2.4, 95% CI: 1.41–3.96) than females. Pharmacists aged ≥ 31 years had higher odds of adequate knowledge than those aged 21–30 years (COR = 3.3, 95% CI: 1.67–6.38), although age was not associated with practices. Married pharmacists had higher odds of adequate knowledge (COR = 4.4, 95% CI: 1.68–11.68) but lower odds of good practices (COR = 0.5, 95% CI: 0.27–0.81) than single pharmacists. Pharmacists with ≥ 6 years of experience had higher odds of adequate knowledge (COR = 4.7, 95% CI: 1.63–13.75). Pharmacy owners had lower odds of good practices than full‐time pharmacists (COR = 0.2, 95% CI: 0.08–0.75), while part‐time pharmacists had higher odds of adequate knowledge (COR = 2.2, 95% CI: 1.10–4.20). After adjustment, male pharmacists remained less likely to have adequate knowledge (AOR = 0.3, 95% CI: 0.14–0.64) but were more likely to demonstrate good practices (AOR = 2.3, 95% CI: 1.27–4.11) than females. Part‐time pharmacists also had higher odds of adequate knowledge than full‐time pharmacists (AOR = 1.5, 95% CI: 1.06–2.21) (Table 6).

**TABLE 6:** Unadjusted and adjusted logistic regression of factors associated with knowledge and practices regarding antibiotics and antibiotic resistance.

Variable	Category	Knowledge				Practice			
		COR	95% CI	AOR	95% CI	COR	95% CI	AOR	95% CI
Gender	Female	1				1			
	Male	0.2	0.12–0.52^∗∗^	0.3	0.14–0.64^∗∗^	2.4	1.41–3.96^∗∗^	2.3	1.27–4.11

Age in years	21–30	1				1			
	≥ 31	3.3	1.67–6.38^∗∗^	1.8	0.79–4.32	0.8	0.45–1.25	—	—

Marital status	Single	1				1			
	Married	4.4	1.68–11.68^∗∗^	1.1	0.33–3.68	0.5	0.27–0.81^∗∗^	0.7	0.33–1.55

Qualification	BPharm	1							
	Higher	NE^†^	NE^†^	NE^†^	NE^†^	1.0	0.41–2.39	—	—

Practising experience	1–5 years	1				1			
	≥ 6 years	4.7	1.63–13.75^∗∗^	3.1	0.90–10.48	1.4	0.77–2.50	—	—

Status at the pharmacy	Full time	1				1			
	Owner	0.9	0.31–2.90	0.2	0.13–2.7	0.2	0.08–0.75^∗^	0.3	0.09–1.32
	Part time	2.2	1.10–4.20	1.5	1.06–2.21	1.4	0.83–2.44	1.7	0.94–3.29

*Note:* Reference category, OR: crude odds ratio.

Abbreviations: AOR, adjusted odds ratio; CI, confidence interval.

^∗^ *p* < 0.05 in both the unadjusted and adjusted model.

^∗∗^ *p* < 0.01 in the both unadjusted and adjusted model.

^†^NE: Not estimable because of sparse data and complete (or quasi‐complete).

### 3.7. Antibiotics Dispensed Without Prescriptions in the Past 12 Months

Figure 4 shows that metronidazole [161/254 (63.4%)], penicillins [158/254 (62.2%)] and macrolides [approximately 122/254 (48.1%)] were the antibiotic classes most frequently dispensed without prescriptions, based on the combined proportions reporting *always* or *often*. Cephalosporins were reported by 114/254 (44.9%) respondents.

**FIGURE 4:**
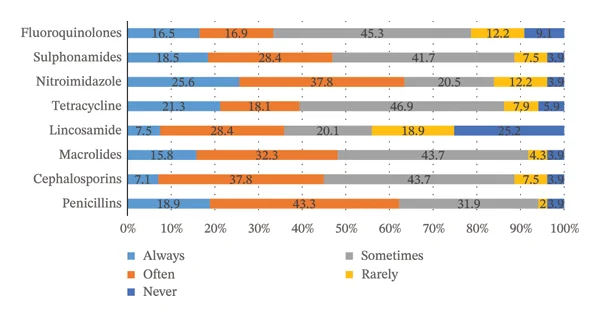
Antibiotics dispensed without prescriptions in the past 12 months (*n* = 254).

## 4. Discussion

This study found that CPs in Lusaka, Zambia, generally demonstrated good knowledge, positive perceptions and good dispensing practices regarding antibiotics and antibiotic resistance. However, variations across individual practice items and participant characteristics suggest that important gaps remain despite the favourable overall findings.

In the current study, 81.0% of CPs demonstrated good knowledge of antibiotic use and antimicrobial resistance. This proportion was higher than the 70.6% reported among CPs in Nigeria [22] and the 37.0% reported in Syria [15], but slightly lower than the 86.0% reported among CPs in Lusaka, Zambia [17]. Differences in knowledge levels across studies may reflect variations in pharmacists’ educational and professional backgrounds, exposure to antimicrobial stewardship activities, study settings and the instruments and cutoff criteria used to classify knowledge. Therefore, comparisons between these proportions should be interpreted cautiously.

Although CPs in our study demonstrated good knowledge of antibiotic use and antibiotic resistance, 13.4% believed antibiotics could be used as first‐line treatment for common colds, coughs, sore throats, and uncomplicated malaria, indicating that misconceptions about appropriate antibiotic use persist despite the generally high level of knowledge observed. Similar knowledge gaps and inappropriate antibiotic dispensing have been reported among community pharmacy personnel in Nigeria [23, 24], Bangladesh [25] and Egypt [26]. Notably, a recent study in Bangladesh reported that 80.1% of community pharmacy staff dispensed nonprescription antibiotics for common cold/influenza [24]. These findings suggest that good overall knowledge may coexist with specific misconceptions regarding appropriate indications for antibiotic use.

In the current study, 85.4% of CPs demonstrated positive perceptions regarding antibioticuse and antimicrobial resistance. This proportion was higher than the 54.3% reported among CPs in southeastern Nigeria [27]. The observed difference may partly reflect variations in the instruments and scoring methods used to assess and classify perceptions across the studies. Most participants (96.5%) agreed that inappropriate antibiotic dispensing can contribute to antibiotic resistance. This finding closely aligns with a study from Nigeria, where 96.8% of CPs similarly recognised inappropriate antibiotic dispensing as a contributor to antibiotic resistance [22]. A study from Libya also reported that more than two‐thirds of CPs recognised that dispensing antibiotics without a prescription contributes to antimicrobial resistance [28]. These findings indicate a generally high levelof awareness among CPs of the contribution of inappropriate antibiotic dispensing to antimicrobial resistance.

In the present study, 61.0% of CPs demonstrated good overall practices regarding antibiotic use and antimicrobial resistance. This proportion was slightly lower than the 66.7% good practice reported among CPs in Lusaka [17], but substantially higher than findings from an earlier study in Lusaka, where only 25.0% demonstrated good practices [19], and from Nigeria, where only 8.7% of CPs had good antibiotic dispensing practices [22]. The relatively favourable practices observed in the present study may reflect increasing awareness of antimicrobial stewardship and appropriate antibiotic use among CPs. However, differences between studies should be interpreted cautiously because of variations in study periods, settings, practice items, scoring systems and cutoff criteria used to classify practice.

Despite the favourable overall practice observed, the finding that nearly 40% pharmacists still had poor practices indicates that important gaps in appropriate antibiotic dispensing remain. Despite the generally favourable overall practice, gaps were evident in specific dispensing behaviours. Nearly two‐thirds (64.6%) of CPs reported that antibiotics were dispensed only with a prescription merely ‘sometimes’, while 53.1% reported only ‘sometimes’ asking about the intended use when antibiotics were requested without a prescription. Similar inconsistencies have been reported in Northern Nigeria, where nonprescription antibiotic dispensing remained common despite pharmacists’ awareness that the practice was prohibited [24]. Likewise, a simulated‐patient study in South Africa found that antibiotics were dispensed without prescriptions in 80% of encounters involving independently owned pharmacies [29]. These findings suggest that awareness of appropriate antibiotic dispensing requirements does not necessarily translate into consistent practice, underscoring the need to strengthen adherence to dispensing standards and appropriate assessment of patients requesting antibiotics.

The self‐reported factors influencing antibiotic dispensing provide important contextual explanations for potentially inappropriate dispensing practices. Notably, 91.7% of CPs perceived weak regulation and limited monitoring as facilitating nonprescription antibiotic dispensing. Similar regulatory concerns have been reported in Sudan, where inadequate monitoring and enforcement of regulations were identified as important contributors to nonprescription antibiotic dispensing [14]. Commercial pressures were also prominent in the present study, with 80.7% of CPs perceiving that refusing customers’ requests for antibiotics could negatively affect their business, while 68.9% perceived antibiotic sales as generating greater profits. Commercial interests and fear of losing customers have similarly been identified as drivers of nonprescription antibiotic dispensing in Sudan and South India [14, 30]. Furthermore, 26.4% of CPs reported that patient pressure could influence them to dispense antibiotics without a prescription. Patient demand has also been identified as an important contributor to inappropriate antibiotic dispensing in community pharmacy settings [14, 24, 30]. Collectively, these findings suggest that dispensing decisions may be shaped by interacting regulatory, commercial and patient‐demand pressures, which may partly explain why adequate knowledge does not always translate into appropriate practice. However, these factors were self‐reported and should be interpreted as perceived influences rather than evidence of causal relationships.

In the current study, male pharmacists had lower odds of adequate knowledge but higher odds of good dispensing practices than females. Previous studies have also reported sex‐related differences in pharmacists’ antibiotic‐related knowledge and practices, although the direction of these associations has not been consistent across settings [24, 28]. This inconsistency may reflect differences in professional experience, training, workplace characteristics and study populations rather than an inherent effect of sex. Part‐time pharmacists in the present study also had higher odds of adequate knowledge than full‐time pharmacists; however, evidence regarding employment status as a determinant of antimicrobial stewardship knowledge remains limited. These findings should therefore be interpreted cautiously and warrant further investigation.

Our study revealed that many CPs frequently dispense antibiotics, including metronidazole, tetracyclines, penicillins, macrolides and fluoroquinolones, without requiring a prescription. Additionally, 91.6% of CPs reported that weak regulatory enforcement and limited monitoring contribute to dispensing antibiotics without a prescription in Zambia. These findings are consistent with those from a study conducted in Nigeria and a review of antimicrobial stewardship in Africa, where similar concerns were reported [22, 31]. There is a need to strengthen regulatory frameworks and increase monitoring of antibiotic dispensing practices in Zambia. Implementing stricter penalties for noncompliance, coupled with regular inspections and audits, could help enforce prescription requirements. Furthermore, educational initiatives targeting both pharmacists and the public on the dangers of antibiotic misuse and resistance are crucial. Encouraging collaboration among healthcare providers, policymakers and regulatory bodies is essential to promote responsible antibiotic use and safeguard public health.

### 4.1. Limitations and Strengths

This study has several limitations. Recall and social desirability biases may have influenced pharmacists’ self‐reported responses. In addition, the study assessed perceived factors influencing antibiotic dispensing; therefore, factors such as weak regulatory enforcement or profit motives should not be interpreted as statistically confirmed determinants. The cross‐sectional design precludes causal inference, and the findings may not be generalisable beyond Lusaka due to possible sampling and self‐selection biases. The practice domain assessed self‐reported antibiotic dispensing behaviours and adherence to professional and regulatory dispensing standards. One item assessed pharmacists’ awareness of the legal requirement to dispense antibiotics only with a valid prescription, reflecting compliance with professional practice expectations. A key strength of the study is the use of multivariable logistic regression to identify factors independently associated with poor dispensing practices while accounting for potential confounding, providing a more robust analysis than descriptive statistics alone. Despite these limitations, the study offers valuable insights into CPs’ knowledge, perceptions and dispensing practices regarding antibiotics and antibiotic resistance.

## 5. Conclusions

CPs in Lusaka demonstrated good knowledge, positive perceptions and generally good dispensing practices regarding antibiotic use and antibiotic resistance. However, gaps in specific dispensing practices remained, highlighting the need for strengthened regulatory oversight and targeted education and training on appropriate antibiotic use and antimicrobial stewardship. Addressing these gaps may further improve dispensing practices and strengthen antimicrobial stewardship in community pharmacy settings.

## Author Contributions

Martin Kampamba, Wayne Chiweza, Jimmy Hangoma and Christabel Mwila Hikaambo contributed to designing the research. Martin Kampamba, Wayne Chiweza and Jimmy Hangoma supervised and coordinated and were responsible for validity. Boris Mwanza, Patricia Mbambara, Ann Silwimbwa, Divine Mbambala and Janipher zulu contributed to material preparation and data collection. Martin Kampamba analysed the data. Jimmy Hangoma, Ann Silwimbwa and Divine Mbambala contributed to the interpretation of the analyses of data. The first draft of the manuscript was written by Martin Kampamba, Wayne Chiweza, Christabel Mwila Hikaambo and Boris Mwanza. All authors commented on the previous versions of the manuscript and approved the final manuscript.

## Funding

This study did not receive any funding.

## Disclosure

Martin Kampamba, the lead author, declares that this article is an honest, accurate and transparent account of the study that is being reported and that no significant elements of the investigation have been omitted. All authors have read and approved the final version of the manuscript.

## Ethics Statement

The protocol for this study was approved by the Ethics Committee from Excellence in Research Ethics and Science Converge (ERES). We obtained informed, written consent from all participants before the study. The participants’ confidentiality and anonymity were strictly maintained.

## Consent

Informed consent from all the participants was taken.

## Conflicts of Interest

The authors declare no conflicts of interest.

## Data Availability

Upon reasonable request, the corresponding author, Martin Kampamba, would provide the data that support the study’s conclusions.

## References

[1] Dhingra S. , Rahman N. A. A. , Peile E. et al., Microbial Resistance Movements: An Overview of Global Public Health Threats Posed by Antimicrobial Resistance, and How Best to Counter, Frontiers in Public Health. (2020) 8, 10.3389/fpubh.2020.535668. PMC7672122 33251170

[2] Lambert M. , van Dijk L. , Benko R. et al., The Role of the Community Pharmacist in Antibiotic Use–A Commentary on Current Status and Future Perspectives, Exploratory Research in Clinical and Social Pharmacy. (2025) 20, 10.1016/j.rcsop.2025.100652. PMC12446664 40980189

[3] Aslam B. , Wang W. , Arshad M. I. et al., Antibiotic Resistance: A Rundown of a Global Crisis, Infection and Drug Resistance. (2018) 11, 1645–1658, 10.2147/idr.s173867. PMC6188119 30349322

[4] Sarwar M. R. , Saqib A. , Iftikhar S. , and Sadiq T. J. I. , Resistance D: KBowledge of Community Pharmacists About Antibiotics, and Their Perceptions and Practices Regarding Antimicrobial Stewardship: A Cross-Sectional Study in Punjab, Pakistaniaat. (2018) 11, 133–145, 10.2147/idr.s148102. PMC5783150 29403295

[5] Rizvi T. , Thompson A. , Williams M. , and Zaidi S. T. R. , Perceptions and Current Practices of Community Pharmacists Regarding Antimicrobial Stewardship in Tasmania, International Journal of Clinical Pharmacy. (2018) 40, no. 5, 1380–1387, 10.1007/s11096-018-0701-1. PMC6208572 30069668

[6] Zhou Z. , Zhao D. , Zhang H. et al., Understanding Parental Self-Medication With Antibiotics Among Parents of Different Nationalities: A Cross-Sectional Study, Global Health Research and Policy. (2021) 6, 1–10, 10.1186/s41256-021-00226-y. PMC8543833 34696814

[7] Llor C. , Bjerrum L. , Molero J. M. et al., Long-Term Effect of a Practice-Based Intervention (HAPPY AUDIT) Aimed at Reducing Antibiotic Prescribing in Patients With Respiratory Tract Infections, Journal of Antimicrobial Chemotherapy. (2018) 73, no. 8, 2215–2222, 10.1093/jac/dky137. 29718420

[8] Al-Halawa D. A. , Seir R. A. , and Qasrawi R. , Antibiotic Resistance Knowledge, Attitudes, and Practices Among Pharmacists: A Cross‐Sectional Study in West Bank, Palestine, Journal of Environmental and Public Health. (2023) 2023, no. 1, 2294048–11, 10.1155/2023/2294048. PMC9902159 36755779

[9] Bassoum O. , Sougou N. M. , Leye Mbacke M. M. et al., Community Pharmacists’ Knowledge, Practices and Perceptions on Antibiotic Use and Resistance: A Cross-Sectional, Self-Administered Questionnaire Survey, Guediawaye and Pikine, Senegal. (2019) .

[10] Essilini A. , Pierre A. , Bocquier A. et al., Community Pharmacists’ Views on Their Current Role and Future Opportunities for Antibiotic Stewardship: A French Qualitative Study, JAC-Antimicrobial Resistance. (2021) 3, no. 3, 10.1093/jacamr/dlab129. PMC8521646 34671729

[11] Haseeb A. , Elrggal M. E. , Bawazir M. S. et al., Knowledge, Attitude, and Perception of Community Pharmacists Towards Antimicrobial Stewardship in Saudi Arabia: A Descriptive Cross-Sectional Study, Saudi Pharmaceutical Journal. (2022) 30, no. 11, 1659–1664, 10.1016/j.jsps.2022.09.010. PMC9715943 36465854

[12] Nagassar R. P. , Carrington A. , Dookeeram D. K. , Daniel K. , and Bridgelal-Nagassar R. J. , Knowledge, Attitudes, and Practices in Antibiotic Dispensing Amongst Pharmacists in Trinidad and Tobago: Exploring a Novel Dichotomy of Antibiotic Laws, Antibiotics. (2023) 12, no. 7, 10.3390/antibiotics12071094. PMC10376728 37508190

[13] Zawahir S. , Le H. T. T. , Nguyen T-A. et al., Inappropriate Supply of Antibiotics for Common Viral Infections by Community Pharmacies in Vietnam: A Standardised Patient Survey, Lancet Regional Health. Western Pacific. (2022) 23, 10.1016/j.lanwpc.2022.100447. PMC9019242 35465041

[14] Salim A. M. and Elgizoli B. , Exploring the Reasons Why Pharmacists Dispense Antibiotics Without Prescriptions in Khartoum State, Sudan, International Journal of Pharmacy Practice. (2017) 25, no. 1, 59–65, 10.1111/ijpp.12317. 27677640

[15] Mansour O. and Al-Kayali R. , Community Pharmacists’ Role in Controlling Bacterial Antibiotic Resistance in Aleppo, Syria, Iranian Journal of Pharmaceutical Research: International Journal of Psychological Research. (2017) 16, no. 4, 1612–1620. PMC5843323 29552070

[16] Allison D. G. , Higginson P. , and Martin S. , Antibiotic Resistance Awareness: A Public Engagement Approach for all Pharmacists, International Journal of Pharmacy Practice. (2017) 25, no. 1, 93–96, 10.1111/ijpp.12287. 27443787

[17] Mufwambi W. , Musuku K. , Hangoma J. , Muzondo N. V. , Mweetwa L. , and Mudenda S. , Community Pharmacists’ Knowledge and Practices Towards Antimicrobial Stewardship: Findings and Implications, JAC-Antimicrobial Resistance. (2024) 6, no. 5, 10.1093/jacamr/dlae176. PMC11521336 39474249

[18] Phiri M. , Shasha L. , Musonda E. , Muhanga M. , and Lemba M. , Choice of Desired Family Size Among Young Women in Zambia: What Matters?, Journal of Population Research. (2023) 40, no. 3, 10.1007/s12546-023-09311-0.

[19] Mudenda S. , Hankombo M. , Saleem Z. et al., Knowledge, Attitude, and Practices of Community Pharmacists on Antibiotic Resistance and Antimicrobial Stewardship in Lusaka, Zambia, 2020, medRxiv.

[20] Chimombe T. , Mudenda S. , Bwalya S. et al., Availability of Antimalarial Medicines in Community Pharmacies of Lusaka District, Zambia: Implications on Compliance to Malaria Treatment Guidelines, Pharmacology & Pharmacy. (2023) 14, no. 4, 85–97, 10.4236/pp.2023.144007.

[21] Kampamba M. , Chawangwana M. , Mwanza B. et al., Assessment of Pharmacists’ Perspectives on State-Run Drug Take-Back Programs and Disposal Practices for Unused and Expired Medications in Zambian Community Pharmacies, Discover Public Health. (2025) 22, no. 1, 10.1186/s12982-025-00941-8.

[22] Akande-Sholabi W. , Oyesiji E. , and Adebisi Y. A. , Antimicrobial Stewardship: Community Pharmacists’ Antibiotic Dispensing Practices, Knowledge, and Perception Regarding Antibiotics and Antibiotic Resistance, Journal of Pharmaceutical Health Services Research. (2023) 14, no. 4, 383–391, 10.1093/jphsr/rmad040.

[23] Akbar Z. , Saleem Z. , Shaukat A. , and Farrukh M. J. , Perception and Practices of Community Pharmacists Towards Antimicrobial Stewardship in Lahore, Pakistan, Journal of Global Antimicrobial Resistance. (2021) 25, 157–161, 10.1016/j.jgar.2021.03.013. 33789207

[24] Abubakar U. and Tangiisuran B. , Knowledge and Practices of Community Pharmacists Towards Non-Prescription Dispensing of Antibiotics in Northern Nigeria, International Journal of Clinical Pharmacy. (2020) 42, no. 2, 756–764, 10.1007/s11096-020-01019-y. 32270378

[25] Sultana S. M. M. , Hossain K. M. , Riha F. A. et al., Understanding the Role of Knowledge and Attitudes of Community Pharmacy Staff in Non-Prescribed Antibiotic Dispensing Practices in Bangladesh: A Cross-Sectional Study, British Medical Journal Open. (2026) 16, no. 1, 10.1136/bmjopen-2025-107698. PMC12778233 41500631

[26] Zakaa El-din M. , Samy F. , Mohamed A. , Hamdy F. , Yasser S. , and Ehab M. , Egyptian Community Pharmacists’ Attitudes and Practices Towards Antibiotic Dispensing and Antibiotic Resistance; A Cross-Sectional Survey in Greater Cairo, Current Medical Research and Opinion. (2019) 35, no. 6, 939–946, 10.1080/03007995.2018.1544119. 30388895

[27] Amorha K. C. , Chima O. M. , Ugochukwu E. J. , and Sabastine R. N. , Assessment of Community Pharmacists’ Knowledge, Perception and Practice Regarding Antimicrobial Stewardship in Ebonyi State, Southeastern Nigeria, African Journal of Biomedical Research. (2024) 27, no. 1, 65–72.

[28] Al-Shami H. A. , Abubakar U. , Hussein M. S. , and Hussin H. F. , Al-Shami SA: ABareness, Practices and Perceptions of Community Pharmacists Towards Antimicrobial Resistance and Antimicrobial Stewardship in Libya: A Cross-Sectional Study, Journal of Pharmaceutical Policy and Practice. (2023) 16, no. 1, 10.1186/s40545-023-00555-y. PMC10028782 36945072

[29] Mokwele R. N. , Schellack N. , Bronkhorst E. , Brink A. J. , Schweickerdt L. , and Godman B. , Using Mystery Shoppers to Determine Practices Pertaining to Antibiotic Dispensing Without a Prescription Among Community Pharmacies in South Africa—A Pilot Survey, JAC-Antimicrobial Resistance. (2022) 4, no. 1, 10.1093/jacamr/dlab196. PMC8826632 35146427

[30] Kumar K. S. , Saranya S. , and Rani N. V. , Community Pharmacists’ Knowledge, Attitude, and Nonprescription Dispensing Practices of Antibiotics: An Explorative Study in a Selected City of South India, Journal of Research in Pharmacy Practice. (2022) 11, no. 2, 51–58, 10.4103/jrpp.jrpp_48_21. PMC9926913 36798105

[31] Gulumbe B. H. , Haruna U. A. , Almazan J. , Ibrahim I. H. , Faggo A. A. , and Bazata A. Y. , Combating the Menace of Antimicrobial Resistance in Africa: A Review on Stewardship, Surveillance and Diagnostic Strategies, Biological Procedures Online. (2022) 24, no. 1, 10.1186/s12575-022-00182-y. PMC9685880 36424530

